# Mechanism of Anti-Salmonella Rabbit Immunoglobulin Adsorption on Polymer Particles

**DOI:** 10.3390/biom13091390

**Published:** 2023-09-15

**Authors:** Paulina Żeliszewska, Monika Wasilewska, Jolanta Szych, Zbigniew Adamczyk

**Affiliations:** 1 Jerzy Haber Institute of Catalysis and Surface Chemistry Polish Academy of Science, Niezapominajek 8, 30-239 Krakow, Poland; monika.wasilewska@ikifp.edu.pl; 2 Biomex Co., Ltd., ul. Friedleina 4-6 lok. 117, 30-009 Krakow, Poland; jszych@gmail.com

**Keywords:** anti-Salmonella immunoglobulin, immunoglobulin adsorption, immunolatex preparation

## Abstract

The adsorption of anti-Salmonella rabbit immunoglobulin (IgaR) on negatively charged polymer particles leading to the formation of immunolatex was studied using various techniques comprising atomic force microscopy (AFM) and laser Doppler velocimetry (LDV). Initially, the basic physicochemical properties of IgaR molecules and the particles, inter alia their electrophoretic mobilities, the zeta potentials and hydrodynamic diameters, were determined under different ionic strengths and pHs. Applying AFM, single immunoglobulin molecules adsorbed on mica were also imaged, which allowed to determine their size. The adsorption of the IgaR molecules on the particles leading to changes in their electrophoretic mobility was monitored in situ using the LDV method. The obtained results were interpreted applying a general electrokinetic model which yielded quantitative information about the molecule coverage on the particles. The obtained immunolatex was thoroughly characterized with respect to its acid–base properties and its stability upon storage. Notably, the developed procedure demonstrated better efficiency compared to commercially applied methods, characterized by a higher immunoglobulin consumption.

## 1. Introduction

The immobilization of protein molecules on particles of a larger size leading to the corona formation offers significant advantages, primarily due to the enhanced stability of such systems in comparison to the native protein solution [1,2,3,4,5,6]. A particularly important role is played by the immunoglobulin corona adsorbed on polymer microparticles referred to as immunolatexes [7,8]. Such conjugates find their application in a variety of tests, including those for bacterial infections such as *E. coli* and *Salmonella* [9,10,11], viral infections such as HIV [12,13,14] and more recently against SARS-CoV-2 [15,16,17,18]. The widespread application of these tests is primarily attributed to their simplicity and remarkably short execution time, typically taking just a few minutes [19]. This characteristics is especially crucial for points-of-care testing, where swift and efficient results are of paramount importance.

However, despite its essential significance, the physicochemical aspects of immunoglobulin (IgG) adsorption on polymer microparticles that lead to the formation of immunolatexes have been only infrequently studied [7,20,21,22,23] because of the limited number of adequate experimental techniques applicalbe under in situ conditions. Serra et al. [20] investigated the adsorption of various IgGs on negatively charged polystyrene microparticles using the electrophoretic mobility and concentration depletion methods. The results showed that the electrophoretic mobility of the particles monotonically increased with the amount of adsorbed IgG and finally reached a plateau value. However, no quantitative interpretation of these results was attempted.

In the work of Sofińska et al. [22], the adsorption of polyclonal IgG on negatively charged polystyrene microparticles was investigated. It was confirmed that the coverage of irreversibly bound protein systematically increased with ionic strength, attaining 2.1 mg m^−2^ for a 0.15 M NaCl solution.

Żeliszewska et al. [23] investigated the adsorption of monoclonal mouse IgG on polystyrene microparticles at different pHs (3.5 and 7.4). Additionally, the interaction of the adsorbed IgG layers with human serum albumin molecules was determined in this study.

However, in neither of these works were the bulk physicochemical properties of the immunoglobulin molecules, such as their electrophoretic mobility, zeta potential, diffusion coefficient and hydrodynamic diameter, determined, which prohibited a quantitative interpretation of the obtained results. Therefore, the mechanism of the antibody monolayer (corona) formation on polystyrene microparticles remains rather obscure. As a result, the commercially implemented procedures of immunolatex preparation are rather intuitive, consisting in a prolonged incubation of the polystyrene microparticles with immunoglobulins of excessive concentrations, followed by a blocking step involving solutions of albumins, commonly bovine serum albumin [7,10,24]. However, such procedures have several drawbacks. They not only increase the consumption of expensive immunoglobulins but also lead to products which contain significant amounts of unadsorbed proteins in the solution. This, in turn, can decrease the stability of the immunolatexes because of the protein aggregation and in consequence, affect the selectivity of assays.

Therefore, the aim of this work was to determine the mechanisms of polyclonal immunoglobulin adsorption on polystyrene particles commonly used as protein carrier particles in commercial tests. In contrast to previous investigations, the bulk physicochemical properties of the immunoglobulin are determined using dynamic light scattering (DLS), laser Doppler velocimetry (LDV) and atomic force microscopy (AFM) methods. This information enabled a quantitative interpretation of the obtained results in terms of a general electrokinetic model. As a result, a valuable insight into the adsorption mechanism of the immunoglobulin was achieved. The practical consequences of these findings are also significant as they facilitate a considerable reduction in the protein consumption needed for immunolatex preparation. Moreover, the elimination of superfluous protein presence in the solution results in a marked improvement of the final product.

## 2. Materials and Methods

### 2.1. Materials

Anti-Salmonella rabbit immunoglobulin (hereafter referred to as IgaR) was supplied by Biomex (Kraków, Poland) as a native solution of the concentration equal to 8500 mg L^−1^. The suspension of polystyrene particles (hereafter referred to as bare latex) applied in these experiments was synthesized in our laboratory according to the Goodwin procedure [25]. The concentration of particles was determined using the densitometry and the dry weight method. It was necessary to dilute the suspension to a concentration of 200 mg L^−1^ before each adsorption experiment.

Ultra-pure water characterized by the electric conductivity of 0.1 µS cm^−1^ was obtained using a Millipore Elix 5 apparatus.

Chemical reagents such as sodium chloride (NaCl) and hydrochloric acid (HCl) were the commercial products of Sigma-Aldrich (Darmstadt, Germany) and were used without further purification.

Ruby muscovite mica obtained from Continental Trade served as a solid substrate for immunoglobulin adsorption measurements. Before each experiment, thin mica sheets of the size 1.5 cm × 1.5 cm were cleaved from bulk mica pieces.

HCl and NaOH solutions were used to adjust the pH during the experiments except for pH 7.4 which was adjusted with PBS buffer.

### 2.2. Experimental Methods

The native IgaR solution was first diluted 10 times to the concentration of 850 mg L^−1^. After filtration using a hydrophilic membrane with low non-specific protein adsorption (<10 µg/cm^2^), the true concentration of this intermediate solution was determined using UV-Vis spectroscopy at 280 nm wavelength. Finally, protein solutions of a desired bulk concentration (typically 0.2–5 mg L^−1^) were prepared using a dilution procedure prior to each adsorption experiment.

The diffusion coefficients of the particles and IgaR molecules were measured with DLS using the Zetasizer Nano ZS Malvern instrument (A.P. Instruments, Warsow, Poland). The corresponding hydrodynamic diameters were calculated using the Stokes–Einstein relationship. The electrophoretic mobilities were measured using the LDV technique using the same device.

In order to imagine of adsorbed IgG molecules on mica, the atomic force microscope (NT-MDT Solver device (Russia) with the SMENA-B scanning head) was used. All measurements were performed under ambient air conditions in semi-contact mode using high-resolution silicon probes (NT-MDT Etalon, HA NC series) and polysilicon cantilevers with resonance frequencies of 140 kHz ± 14 kHz). Using the AFM scans (1000 per 1000 nm), the size and shape of the adsorbed immunoglobulin molecules were determined.

## 3. Results and Discussion

### 3.1. Physicochemical Characteristics of Polystyrene Particles and Immunoglobulin Molecules

The hydrodynamic diameter of the polystyrene microparticles was equal to 830 ± 12 and 820 ± 10 nm in 10 and 150 mM NaCl solutions at pH 3.5, respectively. At a pH of 7.4, the hydrodynamic diameter remained practically the same within experimental error bounds. These results indicate that the particle suspension was stable under the conditions applied in the immunoglobulin adsorption experiments. The zeta potentials of the particles and the protein molecules were calculated from the Henry formula [26] using the LDV electrophoretic mobility data

(1) $$\zeta =\frac{\eta }{\varepsilon f_{H}\left(\kappa d_{H}\right)}\mu_{e}$$

where: *η* is the dynamic viscosity of the electrolyte solution, $\mu_{e}$ is the electrophoretic mobility, *ε* is the dielectric permittivity of the solution, $d_{H}$ is the hydrodynamic diameter, $f_{H}(\kappa d_{H})$ is the Henry function, $\kappa^{-1}={\left(\varepsilon kT/2e^{2}I\right)}^{1/2}$ is the electric double-layer thickness, *k* is the Boltzmann constant, *T* is the absolute temperature, *e* is the elementary charge and *I* is the ionic strength of the electrolyte.

It is interesting to mention that the Henry function approaches the constant value of 2/3 for $\kappa d_{H}$ < 1 (this corresponds to the Hueckel zeta potential limit [27]) and approaches unity for $\kappa d_{H}$ >> 1, attaining the Smoluchowski limit [28]. In the latter case, Equation (1) is valid for an arbitrary zeta potential.

The electrokinetic charge density of the particles was calculated from the Gouy–Chapman relationship valid for a symmetric 1:1 electrolyte

(2) $$\sigma_{0}={\left(8\varepsilon kTn_{b}\right)}^{1/2}\sin h\left(\frac{e\zeta_{i}}{2kT}\right)$$

where: *σ*_0_ is the electrokinetic charge density of the particles, expressed for the sake of convenience as the number of elementary charges per square nm, and *n_b_* is the number concentration of the salt ions in the solution (NaCl). 

The obtained results are collected in Table 1. One can notice that the zeta potential of the particles attains a low value of −100 mV at pH 3.5 and in 10 mM NaCl, which corresponds to the charge density of −0.26 e nm^−2^, that is, one negative charge per 4 nm^2^. At pH 7.4 and in 150 mM NaCl, the particle zeta potential increases to −57 mV, which corresponds to one negative charge per 2.5 nm^2^. The increase in the negative charge density is caused by the increased capacity of the electric double-layer at the particle/electrolyte interface for higher NaCl concentrations.

**Figure 1:**
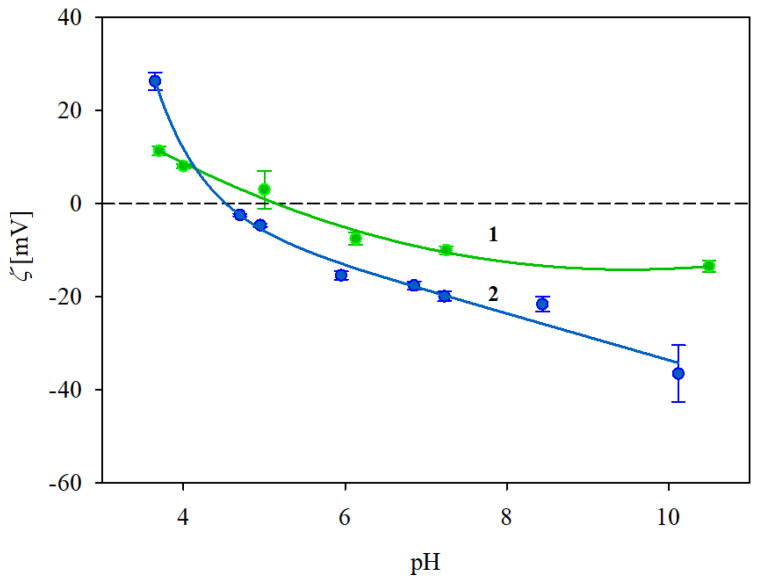
Dependence of the zeta potential of immunoglobulin molecules (IgaR) on pH, determined using the LDV method. The points denote experimental results obtained for 1. (●) 150 mM NaCl, 2. (●) 10 mM NaCl. The solid lines are a guide for the eyes. The dashed line shows the zero value of the zeta potential.

In an analogous way, the dependence of the zeta potential of the IgaR molecules on pH was determined by electrophoretic mobility measurements using Equation (1). The obtained results for 10 and 150 mM NaCl solution concentrations, respectively, are plotted in Figure 1. As can be seen, the protein zeta potential was positive, equal to 26 and 10 mV at pH 3.5 for the 10 and 150 mM NaCl solutions. However, the zeta potential abruptly decreased with the pH becoming negative for pHs higher than 5. In consequence, at pH 7.4, it was equal to −20 and −10 mV at pH 3.5 for the 10 and 150 mM NaCl solutions, respectively (see Table 1).

**Figure 2:**
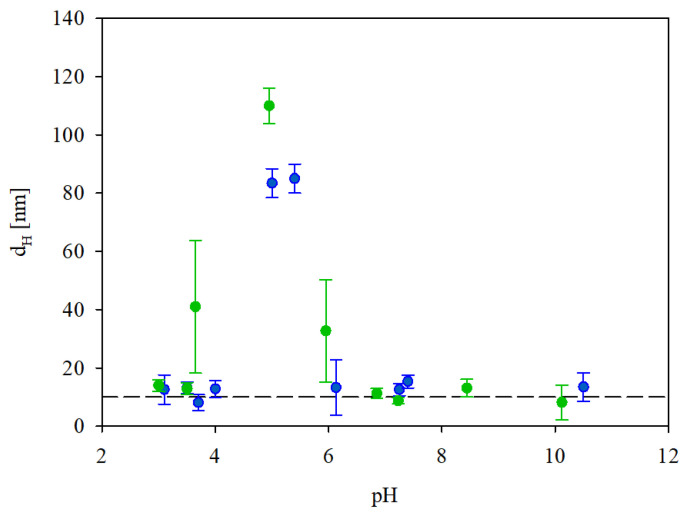
Dependence of the hydrodynamic diameter of IgaR molecules on pH. The points denote experimental results obtained for (●) 10 mM NaCl, (●) 150 mM NaCl. The dashed line shows constant value of *d_H_* equal to 14 nm.

In order to determine the stability of the IgaR solutions, their hydrodynamic diameter was determined as a function of pH using the DLS measurements, applying the Stokes–Einstein relationship. The results shown in Figure 2 indicate that the protein solutions were only stable at pHs up to four and at pH higher than five. Thus, their hydrodynamic diameter was equal to 12 nm at pHs up to 4, 83 nm at pH 5 and 13 nm at pH 6–10. It is worth mentioning that the pH range of protein instability correlates with their zeta potential (see Figure 1), which was practically negligible for pHs between 4 and 5.

**Figure 3:**
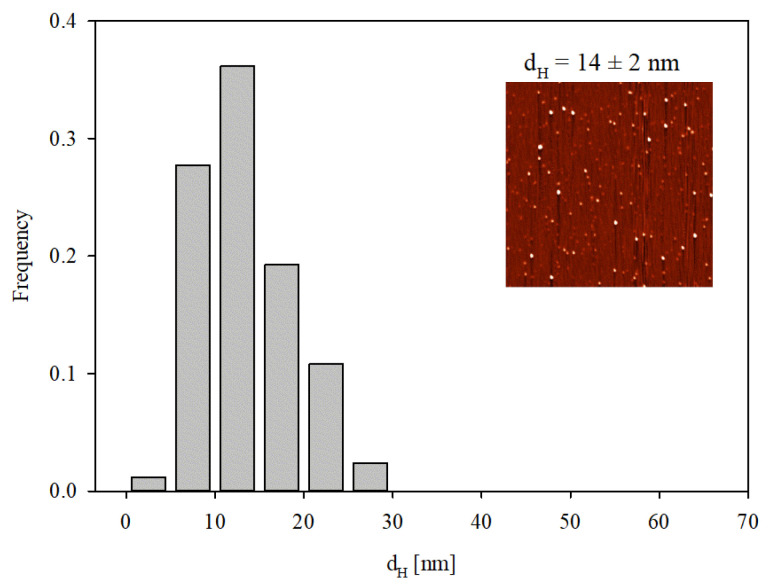
Histogram of IgaR molecule size determined with AFM imaging. The average molecule size was equal to 14 ± 2 nm. The inset shows the molecule layer on mica adsorbed under diffusion-controlled conditions: pH 3.5, 150 mM NaCl, adsorption time 5 min (micrograph size 2 µm × 2 µm).

Given that DLS only yields indirect information about the protein molecule size, supplementary experiments were performed where the adsorption of IgaR molecules on mica was investigated using the AFM method previously applied for fibrinogen [29] and albumins [30]. Because of the molecularly smooth (typical root mean square factor of 0.1 nm) and electrostatically homogeneous surface of mica [31], this method enabled the detection of single molecules and in consequence, the determination of their size and surface concentration. The adsorption was carried out under diffusion transport from a protein solution of 0.5 mg L^−1^ in order to minimize their bulk aggregation. Considering the zeta potential data shown in Figure 1, the IgaR adsorption experiments were carried out at pH 3.5 and in 150 mM NaCl, where the molecules exhibited a positive zeta potential. Under these conditions, the zeta potential of mica was equal to −30 mV. An AFM micrograph of the IgaR molecules adsorbed on mica is shown in the inset in Figure 3. One can observe that the average distance between the molecules is larger than their dimensions, which minimizes the tip convolution artifact that may appear at higher protein surface concentrations.

A qualitative analysis of these micrographs indicated that the molecules exhibit a regular quasi-spherical shape with a relatively small size spread. The size distribution of the molecules was quantitatively determined by measuring their dimensions in two orthogonal directions and taking an average value. The size histogram obtained in this way considering ca. 100 individual molecules is shown in Figure 3. The average size of IgaR molecules determined from the histogram was equal to 14 ± 2 nm, which agrees fairly well with the hydrodynamic molecule diameter derived with DLS at this pH.

### 3.2. Immunoglobulin Adsorption on Polystyrene Microparticles

After establishing the basic physicochemical properties of the microparticles and IgaR molecules comprising their stability, the adsorption experiments were performed according to the procedure previously applied in Refs. [22,23]. Briefly, a protein solution in NaCl of a fixed concentration and pH was mixed over the time of 15 min with an equal volume of the polystyrene microparticle suspension kept at the same pH and NaCl concentration. The bulk concentration of the polystyrene microparticles in the suspension prior to mixing was kept constant and equal to 200 mg L^−1^, whereas the protein concentration was varied in a discrete manner from 0.2 to 5 mg L^−1^. After completing an adsorption run, the electrophoretic mobility of the microparticles with the protein corona was measured using the LDV method, and the corresponding zeta potential was calculated using the Smoluchowski equation.

It should be mentioned that the relaxation time of the protein corona formation $t_{c}$ is independent of the protein concentration and can be calculated as

(3) $$t_{c}=d_{H}^{2}\frac{{\left[{\left(\Phi_{V}/\Phi_{mx}\right)}^{-1/3}-1\right]}^{2}}{4\overset{-}{D_{}}}$$

where $d_{H}$ is the hydrodynamic diameter of the microparticles, $\Phi_{V}$ is their bulk volume fraction, $\Phi_{mx}$ is the maximum volume fraction equal to 0.62 for a quasi-random packing of spheres and $\overset{-}{D_{}}$ is the mutual diffusion coefficient of the protein molecule relative to the particle.

Because of the much larger size of the polystyrene microparticles compared to the protein size, $\overset{-}{D_{a}}$ was practically equal to the diffusion coefficient of the IgaR molecules in the bulk, that is, 4 × 10^−11^ m^2^⋅s^−1^. Considering the values *d_H_* = 820 nm and $\Phi_{V}$ = 10^−4^ (this corresponds to the bulk polystyrene microparticle concentration in the mixture of 100 mg L^−1^), one obtains $t_{c}$ = 1 s from Equation (3), which indicates that the adsorption time was sufficient in order to form the IgaR corona at the particles.

The primary results derived from these experiments were the dependencies of the zeta potential of the particles on the IgaR bulk concentration. However, for practical purposes, it is also advantageous to express these results as the dependence of the zeta potential on the mass coverage of the protein in mg m^−2^, calculated from the formula [32,33,34]

(4) $$\Gamma =\left(\frac{\rho_{p_{}^{}}d_{H}}{6}\right)\frac{c_{p}}{c_{l}}$$

where $\rho_{p_{}^{}}$ is the particle density equal to 1.05 g cm^−3^, *c_p_* and *c_l_* are the protein and the particle concentrations in the suspension.

Under our experimental conditions, considering that $d_{H}$ = 820 nm and *c_l_* = 100 mg L^−1^, one can calculate from Equation (4) that Γ = 1.4 mg m^−2^ for the protein concentration of 1 mg L^−1^.

**Figure 4:**
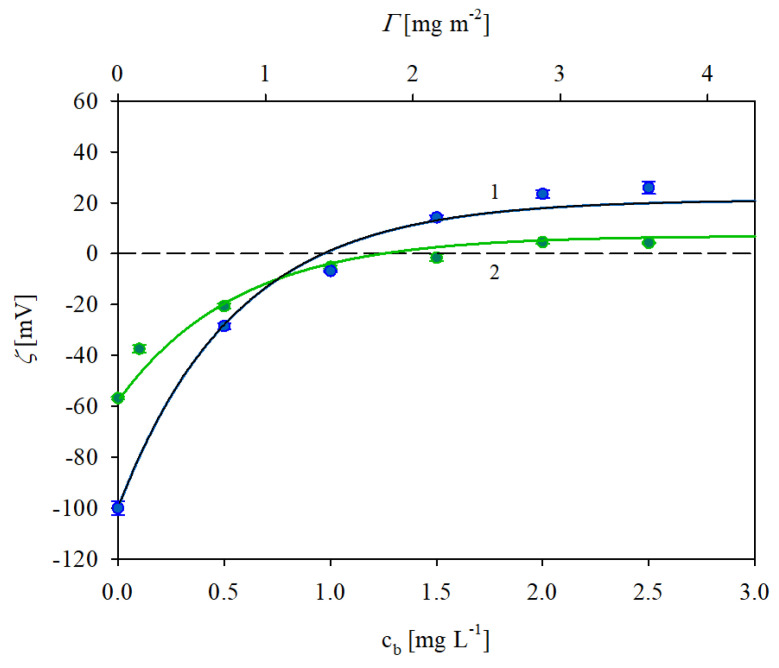
Zeta potential of polystyrene microparticles vs. the IgaR initial concentration in the suspension (the upper horizontal axis shows the nominal molecule coverage in mg m^−2^ corresponding to this concentration). The points denote experimental results obtained for pH 3.5 and 1. (●) 10 mM NaCl, 2. (●) 150 mM NaCl. The solid lines show the theoretical results calculated from the 3D electrokinetic model (Equations (5) and (6)). The dashed line shows the zero value of the zeta potential.

In Figure 4 the dependencies of the zeta potential of the polystyrene microparticles on the bulk IgaR concentration (lower axis) and the mass coverage calculated from Equation (4) (upper axis) obtained at pH 3.5 are shown. It can be observed that the initially negative zeta potential rapidly increased with the protein coverage and became positive for c*_l_* higher than 1 mg L^−1^ (this corresponds to Γ higher than 1.4 mg m^−2^ as estimated above). For a still higher protein coverage, the zeta potential became positive and finally attained a plateau value of 21 mV. Analogous results were obtained for the 150 mM NaCl concentration with the plateau value of the zeta potential equal to 6 mV. In both cases the plateau values of the zeta potential corresponded to Γ equal to ca 2.8 mg m^−2^.

It is worth underlining that the results shown in Figure 4 can be adequately interpreted in terms of the attractive electrostatic interactions among the positively charged IgaR molecules and the polystyrene microparticles exhibiting a high negative charge density at pH 3.5; see Table 1.

The results shown in Figure 4 were interpreted in terms of the general electrokinetic model developed in Refs. [35,36] and previously applied for albumin [30] and fibrinogen [29]. The following formula for the zeta potential of polystyrene microparticles covered by a protein layer denoted by ζ(Θ) was derived.

(5) $$\zeta (\Theta )=F_{i}(\Theta )\zeta_{i}+F_{p}(\Theta )\zeta_{p}$$

where Θ is the dimensionless protein coverage, $\zeta_{i}$ is the zeta potential of the polystyrene microparticles, $\zeta_{p}$ is the zeta potential of the protein in the bulk and $F_{i}\left(\Theta \right),\;F_{p}\left(\Theta \right)$ are the dimensionless functions. The *F_i_* function describes the damping of the flow near the particle surface by the adsorbed molecule layer and the *F_p_* function characterizes the contribution to the zeta potential stemming from the electric double-layer surrounding the molecules. These functions were calculated in Ref. [36] by applying the multipole expansion method.

The dimensionless coverage occurring in Equation (5) is connected with the mass coverage via the following constitutive dependence:

(6) $$\Theta =S_{g}\left(\frac{{Ν}_{av}}{M_{w}}\right)\Gamma$$

where *S_g_* is the characteristic cross-section area of the IgaR molecule, ${Ν}_{av}$ is the Avogadro number and $M_{w}$ is the protein molar mass.

One can observe in Figure 4 that the theoretical results calculated from Equations (5) and (6) adequately reflected the experimental data obtained for pH 3.5 considering that $M_{w}$ = 150 kDa (kg mol^−1^) and assuming that the effective cross-section area of the IgG molecule was equal to 55 nm^2^. This value is close to the characteristic cross-section area derived from the crystallo-hydrodynamic model by Carasco et al. [37] for the perpendicular orientation of adsorbed immunoglobulin molecules which was equal to 51 nm^2^.

**Figure 5:**
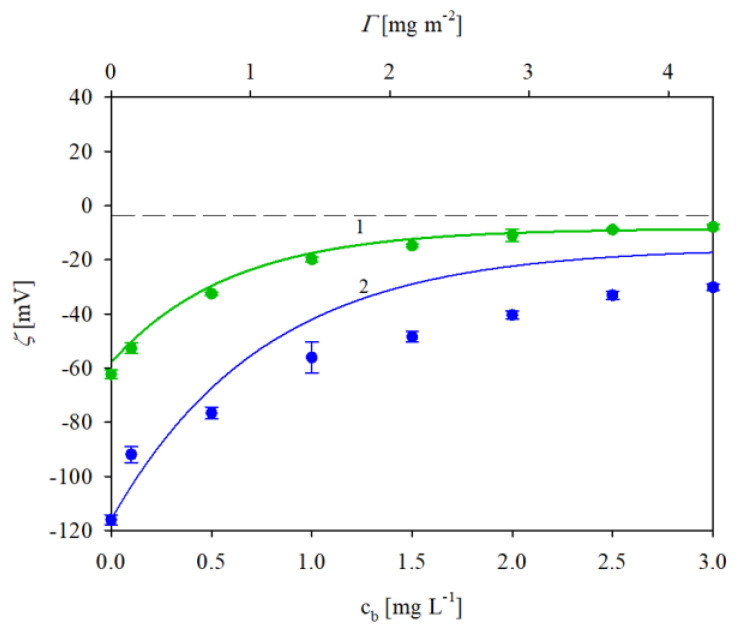
Zeta potential of polystyrene microparticles vs. the IgaR initial concentration in the suspension (the upper horizontal axis shows the nominal molecule coverage in mg m^−2^ corresponding to this concentration). The points denote experimental results obtained at pH 7.4 and in 1. (●) 150 mM NaCl, 2. (●) 10 mM NaCl. The solid lines show the theoretical results calculated from the 3D electrokinetic model (Equations (5) and (6)). The dashed line shows the zero value of the zeta potential.

Analogous results were obtained for IgaR molecule adsorption at pH 7.4; see Figure 5. As before (for pH 3.5), the polystyrene microparticle zeta potential increased with the protein coverage. However, in this case, the plateau zeta potential values remained negative and equal to −10 and −40 mV for NaCl concentrations of 150 and 10 mM, respectively. As previously discussed, the experimental results obtained for the NaCl concentration of 150 mM were quantitatively described by the electrokinetic model; see the solid line 1 in Figure 5. This confirms the efficient adsorption of the protein, whose maximum coverage attained the value of ca. 2.8 mg m^−2^. It should be mentioned, however, that this behavior is rather unexpected from the point of view of the mean-field electrostatic interaction theory because the protein molecules exhibiting a negative zeta potential adsorbed on negatively charged particles. This discrepancy, previously observed for fibrinogen [29], can be explained by the heterogeneous charge distribution over the immunoglobulin molecule characterized by the presence of positive patches located within the antigen binding Fab domain [38,39]. This hypothesis is confirmed by the fact that for the lower NaCl concentration of 10 mM, where the zeta potentials of the particles and the protein were more negative, the experimental results were markedly below the theoretical line 2 indicating that the IgaR adsorption was less efficient. One can also suppose that the charge regulation mechanism studied in Refs. [40,41] can explain the anomalous IgaR molecule adsorption on negatively charged polymer particles at pHs above its isoelectric point. Extensive theoretical modeling performed in these works showed that this effect was significant for globular proteins such as lysozyme and human serum albumin. Unfortunately, at the present time, theoretical results for immunoglobulin molecules are not available.

The results presented in this section confirm that the above method can be successfully applied to prepare polymer particles covered with immunoglobulin (IgaR) characterized by a precisely controlled coverage and zeta potential. An additional advantage of this method consists of the elimination of the presence of superfluous protein presence in the solution, which may decrease the particle stability. Therefore, in our further investigations, the particles covered by the IgaR corona characterized by a concentration equal to 2.8 mg m^−2^ formed at pH 3.5 or 7.4, hereafter referred to as the immunolatexes, were used.

### 3.3. Stability and Electrokinetic Characteristics of the IgGaR Immunolatex

**Figure 6:**
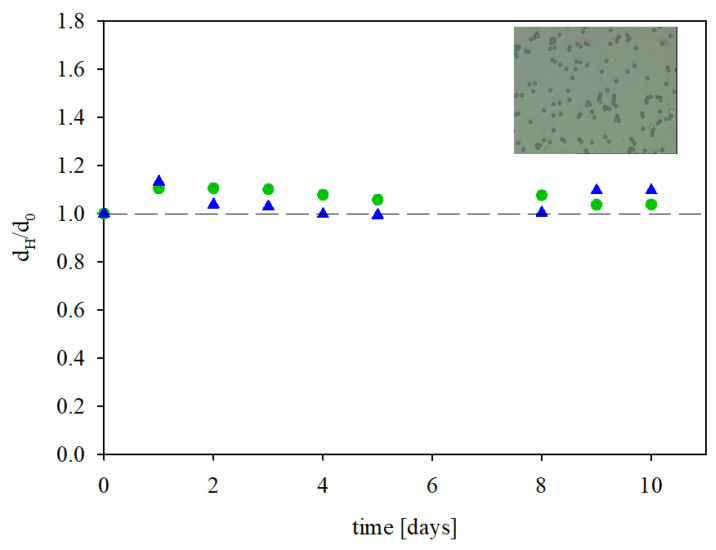
The dependence of the normalized hydrodynamic diameter of the IgaR immunolatex on the storage time, (●) pH 3.5, 150 mM NaCl; (▲) pH 7.4, 150 mM PBS. The inset shows the layer of the immunolatex on mica imaged with optical microscopy (micrograph size 45 µm × 35 µm). The dashed line shows the reference value of unity.

The stability of the immunolatexes was established in experiments where their hydrodynamic diameter and zeta potential were determined over a prolonged time period under a fixed ionic strength and pH. The obtained results, shown in Figure 6 as the dependence of the normalized hydrodynamic diameter *d_H_*/*d_Ho_* (where *d_Ho_* is the initial hydrodynamic diameter) on time, show that these parameters did not change over time for up to 10 days. This fact confirmed their stability and allowed for precise electrokinetic characteristics of the immunolatex in experiments which were usually completed within one hour.

**Figure 7:**
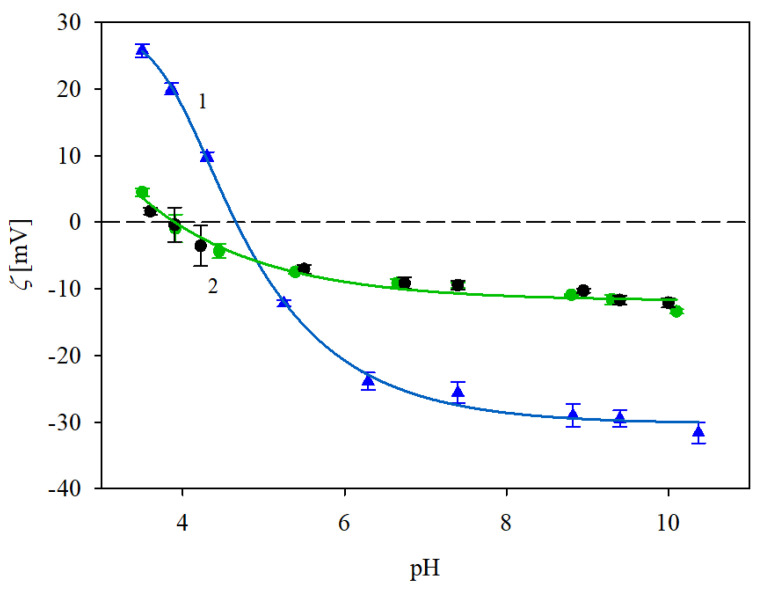
Zeta potential of the immunolatexes (IgaR coverage of 2.8 mg m^−2^) vs. pH. The points denote experimental results acquired with (▲) 10 mM NaCl, (●) 150 mM NaCl, protein adsorption on polystyrene microparticles being carried out at pH 3.5; (●) 150 mM NaCl, protein adsorption at pH 7.4. The solid lines 1 and 2 are guides for the eyes. The dashed line shows the zero value of the zeta potential.

The electrokinetic characteristics of the immunolatexes were acquired as follows: initially, an IgaR layer of coverage equal to 2.8 mg m^−2^ was adsorbed on the polystyrene microparticles at pH 3.5 and in a NaCl concentration of either 10 or 150 mM. Subsequently, the pH was increased by adding NaOH in a stepwise manner. After each pH adjustment, the system was allowed to stabilize for a few minutes. Afterward, the electrophoretic mobility of the immunolatex was measured and the zeta potential was determined with the Smoluchowski equation. Analogous measurements were carried out for the immunolatex prepared by the adsorption of the IgaR molecules at pH 7.4, stabilized by the PBS buffer. The results of these measurements are presented in Figure 7. As can be seen, the zeta potential of the immunolatexes (prepared at pH 3.5) was equal to 25 and 5 mV, for the 10 and 150 mM NaCl concentrations, respectively, at the same pH of 3.5. Their zeta potential abruptly decreased for higher pHs, changing its sign at a pH of about 4–5 for the 150 and 10 mM NaCl solutions, respectively. Interestingly, the immunolatex where the IgaR molecule adsorption was carried out at pH 7.4 (depicted by black circles in Figure 7) exhibited an almost identical electrokinetic behavior.

**Figure 8:**
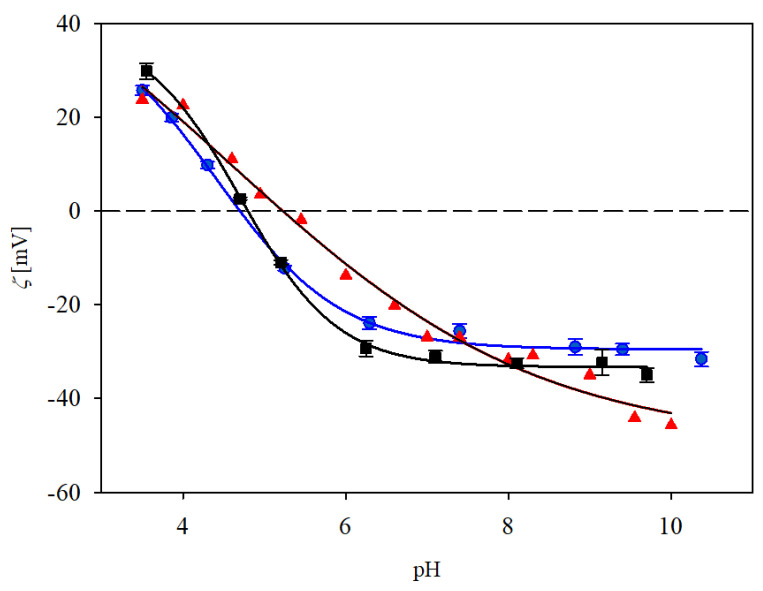
Zeta potential of various immunolatexes vs. pH determined at 10 mM NaCl. (▲) previous results for mouse monoclonal IgGs, (●) the IgaR immunolatex (this work, immunoglobulin coverage 2.8 mg m^−2^, adsorption at pH 3.5), (■) the SAL latex commercial product of BIOMEX [24]. The solid lines are guides for the eyes. The dashed line shows the zero value of the zeta potential.

The electrokinetic properties of the immunolatex obtained in our work were compared with the ones previously determined for monoclonal immunoglobulin (anti-fibrinogen) [23] and the commercial immunolatex of BIOMEX, previously studied in Ref. [24] and referred to as SAL. This latex was prepared by the incubation of the polymer particles with IgaR solution of the concentration a 40 times larger than in our study. The results shown in Figure 8 indicate that the dependences of the zeta potential of our immunolatexes of concentrations was practically identical.

## 4. Conclusions

Physicochemical characteristics of the anti-Salmonella immunoglobulin comprising its molecule size, zeta potential and the stability of the solutions under different pHs and ionic strengths were acquired.

These experimental data enabled a quantitative interpretation of the protein adsorption on polystyrene microparticles in terms of a general electrokinetic model. It was confirmed that the adsorption was irreversible and controlled by electrostatic interactions. Furthermore, the electrophoretic mobility (LDV) method applied in these experiments enabled to directly determine the immunoglobulin coverage on the particles under in situ conditions.

Exploiting these results, a reliable method for the preparation of immunolatexes characterized by well-controlled protein activity and electrokinetic properties was developed. In contrast to commercial procedures, the use of immunoglobulin was minimized, and the albumin blocking step was entirely discarded by keeping the immunolatex stability intact.

## Figures and Tables

**Table 1:** Electrophoretic mobilities *µ_e_*, zeta potentials *ζ_i_*, *ζ_p_* of the polystyrene particles and the IgaR immunoglobulin molecules at different NaCl concentrations and pHs.

pH	NaCl Concentration [mM]	Polystyrene Microparticles			Immunoglobulin IgaR	
		*µ_e_* [µm cm/Vs]	*ζ_i_* [mV]	*σ*_0_ [e nm^−2^]	*µ_e_* [µm cm/Vs]	*ζ_p_* [mV]
3.5	10	−7.8 ± 0.2	−100 ± 3 −101 ± 3 *	−0.26	1.4 ± 0.1	26 ± 1 *
	150	−4.2 ± 0.1	−54 ±1 −54 ± 1 *	−0.36	0.59 ± 0.1	10 ± 2 *
7.4	10	−9.0 ± 0.3	−116 ± 2 −117 ± 2 *	−0.34	−1.1 ± 0.2	−20 ± 3 *
	150	−4.5 ± 0.2	−57 ± 2 −58 ± 2 *	−0.39	−0.52 ± 0.2	−10 ± 1 *

* calculated from the Henry model.

## Data Availability

The data presented in this study are available on request from the corresponding author.
